# Electroacupuncture and vagus nerve stimulation are emerging therapies for hyperemesis gravidarum management

**DOI:** 10.3389/fneur.2025.1615635

**Published:** 2025-10-15

**Authors:** Shike Zhang, Hui He, Xinyu Li, Yi Guo

**Affiliations:** ^1^Research Center of Experimental Acupuncture, Tianjin University of Traditional Chinese Medicine, Tianjin, China; ^2^National Clinical Research Center for Chinese Medicine Acupuncture and Moxibustion, Tianjin, China; ^3^Department of Reproductive Medicine, First Affiliated Hospital of Zhengzhou University, Zhengzhou, China; ^4^College of Traditional Chinese Medicine, Tianjin University of Traditional Chinese Medicine, Tianjin, China

**Keywords:** hyperemesis gravidarum, nausea and vomiting of pregnancy, electroacupuncture, vagus nerve stimulation, acupuncture, growth and differentiation factor 15

## Abstract

Hyperemesis gravidarum (HG), an extreme form of nausea and vomiting during pregnancy (NVP), significantly impairs the quality of life of affected individuals. This review examines the multifactorial etiology of the HG and evaluates the efficacy of non-traditional therapeutic approaches, namely, vagus nerve stimulation (VNS) and electroacupuncture. By synthesizing a comprehensive body of literature, we highlight the neurophysiological mechanisms underlying these therapies and propose a novel model for the HG management. The model focuses on vagus nerve modulation through targeted stimulation at the auricular and cervical points, suggesting a promising avenue for effective and safe treatment strategies against the NVP/HG.

## 1 Introduction

Hyperemesis gravidarum (HG) encompasses a range of severe symptoms associated with nausea and vomiting during pregnancy (NVP) (1). Nausea, which is characterized by a sensation of upper abdominal discomfort and imminent vomiting, can be accompanied by symptoms associated with vagal nerve excitation. Vomiting can be defined as a phenomenon in which the contents of the stomach or duodenum are forced out of the body through the esophagus or mouth by strong contraction of the stomach, diaphragm, and other abdominal muscles (2). According to a recent international consensus, signs of dehydration or metabolic disorders (weight loss, electrolyte deficiency, or undernourishment) are considered helpful in defining the HG (3). In pregnant women, NVP symptoms commonly appear before 9 weeks of conception and disappear after 20 weeks of conception (4). However, the NVP affects up to 70% of pregnant women (5). Albeit the HG is a pathological form of the NVP that reportedly occurs in 0.3% to 3% of gravidas (6). Approximately 10% of gravidas with the HG are affected during gestation (7). In addition, the HG is the most common reason for hospital admission in the first trimester of pregnancy and is associated with a substantial estimated economic burden (8). In addition to considerable maternal morbidity during pregnancy, the HG poses a significant risk to the long-term health of both mothers and children (9–11).

Growth and Differentiation Factor 15 (GDF15), a placenta-derived protein, is associated with NVP symptoms (12). As a nonspecific blood biomarker of the HG, GDF15 crosses the blood-brain barrier and binds to the glial cell-derived neurotrophic factor family receptor α-like (GFRAL) receptor in the area postrema (AP) and nucleus of the solitary tract (NTS) of the brainstem, which influences food intake, nausea, body weight, and insulin sensitivity (13, 14). Because metformin increases circulating GDF15 levels, its use before pregnancy lowers the risk of severe NVP and HG (15, 16). Activated AP/NTS neurons project to the hypothalamic arcuate nucleus (ARC), where vagal efferent signaling suppresses gastric motility (17). Furthermore, 5-HT plays a critical role in the body's regulation of vomiting, and its receptor, 5-hydroxytryptamine receptor (5-HT3R), is present in both vagal afferent fibers and AP/NTS neurons in the hindbrain terminal (18). 5-HT3 receptor antagonists have been approved for the NVP/HG management, including ondansetron by the Food and Drug Administration (FDA) (19, 20). First-trimester exposure correlates with a slightly increased risk of oral clefts (21). Although newer antiemetics show improved safety profiles, teratogenicity concerns persist, compromising treatment adherence (6, 22). Current evidence for HG therapies remains limited (23, 24), underscoring the need for novel non-pharmacological interventions (5).

Despite these challenges, emerging evidence has supported the use of alternative approaches. A systematic review and meta-analysis found that acupuncture alleviated the symptoms in pregnant women with NVP (25). Another meta-analysis suggested that acupuncture is effective in treating the HG (26). From a neurophysiological perspective, contemporary research suggests that acupuncture may alleviate the NVP/HG by regulating the activity of the vagus nerve, highlighting the pivotal role of vagal regulation in the treatment of the NVP/HG. This assertion is supported by the results of recent neurophysiological studies. For example, acupuncture can increase parasympathetic nerve activation (27). Low-frequency electroacupuncture at acupoints in the lower limbs can decrease sympathetic nerve activity (28). Additionally, the anti-inflammatory responses to acupuncture stimulation require activation of the vagus nerve pathway (29). Owing to the unique significance of the vagus nerve in the human body, an increasing number of studies have been conducted on vagus nerve stimulation (VNS) (30). Anatomically, VNS mainly regulates the vagus nerve in the cervical or auricular region. In this review, we systematically examined the existing literature on the use of acupuncture and related techniques in the management of the NVP/HG. Based on a systematic literature review, we propose that electroacupuncture at auricular/cervical points, by leveraging VNS, can potentially offer a novel treatment avenue for the NVP/HG.

## 2 Pathogenesis

Although NVP and HG are common conception disorders, studies of their pathogenesis are lacking. Related literature has outlined the historical presuppositions on the nosogenesis of the NVP and HG, including hormonal factors, *Helicobacter pylori* (*H. pylori)*, gastrointestinal dysmotility, placenta-related factors, psychosocial factors, and newly discovered genetic factors (5, 31, 32).

### 2.1 Hormonal factors

Elevated human chorionic gonadotropin (HCG) levels during the first trimester of pregnancy have been implicated in the pathogenesis of the NVP (22). Clinical evidence from 8,195 women demonstrated a significant correlation between the HCG concentration and the NVP severity (33). A meta-analysis indicated that the HG in pregnant women is associated with elevated HCG serum levels (34). High levels of HCG may result in disturbed gastrointestinal motility, delayed gastric emptying, and increased nausea and vomiting. Regarding estrogen, a positive correlation between estradiol levels and NVP intensity has been documented (35). Consistently, two independent seroepidemiological studies revealed significantly higher estradiol concentrations in HG patients compared to controls (36, 37). Notably, the peak levels of sex hormones did not temporally align with the onset of HG symptoms. Progesterone involvement was supported by a recent meta-analysis that identified associations between elevated serum progesterone concentrations and HG in two studies (38–40). However, contradictory evidence suggests that high progesterone levels—whether endogenous or exogenous—may not independently drive the HG pathogenesis (41). Elevated serum GDF15 levels were documented in HG patients at 12 gestational weeks, whereas no significant difference in HCG levels was observed between cases and controls (42). Furthermore, while circulating the GDF15 demonstrated a strong positive correlation with the HCG and was associated with second-trimester vomiting severity and antiemetic use, HCG levels showed no significant elevation in women experiencing second-trimester vomiting (43). Consequently, research must prioritize elucidating the molecular pathogenesis of the NVP and HG over traditional hormone HCG-centric paradigms.

### 2.2 Gastrointestinal dysfunction (abnormal gastrointestinal motility)

Abnormal upper gastrointestinal motility is assumed to be a cause of the NVP and HG (44). Numerous studies have found that women with NVP may have a slow gastric wave rhythm, which may be attributed to elevated levels of endogenous estrogen and progesterone (45–47). Furthermore, Owyang et al. found that progesterone and estrogen may make individuals susceptible to disturbances in gastric slow-wave rhythm and inevitably affect peristalsis and stomach emptying, resulting in NVP (48, 49). In addition, GDF15-mediated gastric emptying delay represents a pathophysiological mechanism contributing to nausea (5). In *ex vivo* rodent gastric smooth muscle, where GDF15 receptors were detected, exposure to GDF15 caused depolarization and increased mechanical activation (50).

### 2.3 Placenta-related factors and emerging factors identified by genetics

Several studies have revealed a close association between the HG or NVP and factors such as placental weight, hormones produced by the placenta, and genes (GDF15, IGFBP7, and PGR) expressed within the placenta (51–53). The placenta is the primary tissue that exhibits elevated levels of GDF15 under normal physiological conditions during prenatal development (54). GDF15 is primarily secreted by the placenta in response to various stressors (55, 56) and interacts with its receptor to mediate nausea-associated behaviors (57). Furthermore, the data show that elevated circulating levels of GDF15 are significantly associated with both second-trimester NVP and HG (42, 43).

Genetic susceptibility is pivotal, with an NVP heritability of 73% (58). Familial aggregation reveals extreme recurrence; sisters of HG patients have a 17-fold elevated risk, and mother-daughter recurrence exceeds 27-fold with maternal history plus two affected daughters (59, 60). Genome-wide association studies (GWAS) identified the placental gene loci GDF15 and IGFBP7 as principal genetic determinants of HG (61). GDF15 has emerged as a paramount genetic risk factor, with placental-derived GDF15 protein activating brainstem nausea pathways via its receptor GFRAL (62). Notably, GWAS signals implicate both GDF15 and its receptor GFRAL, suggesting ligand-receptor co-regulation in HG pathophysiology (61). Insulin-like growth factor binding protein 7 (IGFBP7) is involved in the implantation and decidualization of the pregnant uterus (63). Both GDF15 and IGFBP7 demonstrate synchronous post-implantation upregulation and sustained placental expression, establishing a shared molecular framework for appetite dysregulation and vomiting reflexes during pregnancy (64).

### 2.4 *Helicobacter pylori* infection

A meta-analysis revealed that *H. pylori* infection is associated with an increased likelihood of the HG during pregnancy (65). *Helicobacter pylori* infection is an independent risk factor for vomiting during pregnancy (66). Many case-control studies have shown a significant positive association between the HG and *H. pylori* infection in pregnancy studies (67–69). In addition, a systematic review found that the prevalence of the HG in *H. pylori*-infected pregnant women was higher than in uninfected ones (70). A case-control study of 444 gravidas (148 cases and 296 controls) identified *Helicobacter pylori* as a determinant of the HG (71). Interestingly, in a study on walnut polyphenol extract (WPE) against *H. pylori* infection, GDF15 was identified as one of the key genes significantly upregulated by WPE (72).

### 2.5 Psychosocial factors

Psychological factors are increasingly being recognized as contributors to the NVP and HG. Studies indicate that pregnant women with pre-existing psychological disorders are more susceptible to these conditions (73). Furthermore, adverse psychological states—including depression, anxiety, and stress—have been specifically associated with the HG and/or NVP (74–76). Supporting this, a prospective study by Tan et al. (77) found that 57.4% of HG women met the criteria for depression or experienced significant anxiety. Annagür et al. (78) also reported that anxiety in pregnant women was associated with the pathogenesis of HG, while Koken et al. (79) found a positive correlation between the severity of nausea and vomiting and that of anxiety and depression early in pregnancy. Notably, elevated plasma GDF15 concentrations, implicated in both nausea/vomiting pathways and depressive states (80, 81), may represent a potential biological link between these psychological factors and the development or severity of the HG/NVP.

## 3 Selection of acupuncture points and vagus nerve stimulation for NVP/HG treatment

With a growing focus on treating the NVP, especially the HG, and concerns about the side effects of antiemetic drugs, acupuncture, as a significant constituent of complementary and alternative medicine, has emerged because of its positive curative outcomes, cost-effectiveness, straightforward operation, and minimal adverse reactions, and it provides a wide range of choices for doctors and patients. Modern physiologists have put forward a “neural hypothesis,” proposing that acupuncture can primarily irritate sensory nerves close to the interposing needle underneath the skin, deliver signals to the cerebrum, and produce clinical influence (82). Neiguan (PC6) is located three finger widths (5 cm) above the transverse crease of the wrist and between the palmaris longus tendon and the flexor radialis carpi tendon, whereas Zusanli (ST36) is located at four finger widths (7.2–8 cm) from the lower edge of the patella and one finger width (1.8–2 cm) lateral to the anterior tibial crest (83, 84). In recent years, acupuncture has been increasingly employed to treat the NVP, especially in Asia. Acupressure at the PC6 acupoint has also been proven to be an efficacious non-medicinal intervention to alleviate NVP (85). Acupuncture at PC6 was found to be more effective in alleviating the NVP and HG than placebo acupuncture (86, 87). In both British and Canadian guidelines, PC6 acupressure and acupuncture are considered safe and effective (88, 89). In addition, two meta-analysis studies (90, 91) found that massage at PC6 had some effect on alleviating NVP. A randomized controlled trial arrived at the conclusion that electroacupuncture at PC6 and ST36 is more effective in relieving emesis than the medicinal treatment of antiemetic pharmacotherapy alone (92). However, the American College of Obstetricians and Gynecologists (ACOG) concluded that acupuncture at PC6 currently lacks sufficient medical evidence (93). Therefore, in 2023, Wu et al. (94) published the results of a multicenter, randomized, double-blind, placebo-controlled, and 2 × 2 factorial trial with 352 women in the first trimester with moderate-to-severe NVP from 13 tertiary hospitals in mainland China. This study demonstrated the efficacy of acupuncture and doxylamine-pyridoxine alone in treating moderate and severe NVP. However, doxylamine-pyridoxine was associated with a significantly higher risk of small-for-gestational-age (SGA) births than placebo [odds ratio (OR) 3.8, 95% CI 1.0–14.1]. PC6 and ST36 were chosen as core acupuncture points. This latest clinical study, corroborating the strongest evidence-based medical support for acupuncture in treating NVP, concurs with Mazzone et al.'s (95) viewpoint that acupuncture may alleviate NVP through vagal modulation via local acupoint stimulation. The therapeutic effect of electroacupuncture (EA) at PC6 is achieved through the vagus nerve (VN) (96). EA at ST36 can increase the VN's efferent activity (97). In addition, non-invasive TEA at ST36 is effective in enhancing vagal and suppressing sympathetic activities (98).

Anatomically, the median nerve is located below the PC6 acupoint, which originates from the lateral and medial sides of the brachial plexus and has ventral roots of C5–C7 (lateral) and C7–T1 (medial). Jamigorn et al. (99) found that relieving nausea and vomiting by applying pressure at PC6 irritates the median nerve. Neuroanatomically, the ST36 is primarily located close to the sciatic nerve and its branches. The sciatic nerve is composed of lumbar 4/5 and sacral 1/2/3 nerves. Intriguingly, Zhang et al. (100) found that EA at ST36 can exert protective effects via the sciatic nerve and cervical NV. Specifically, acupuncture points on the abdomen can stimulate the sympathetic nervous system of the corresponding segment to inhibit stomach movement, whereas those on the limbs can stimulate the vagus nerve to promote gastric movement (101, 102). When the former is stimulated, sympathetic nerves are activated to restrain gastric motility (103). EA at PC6 in the upper limb can remarkably affect C-Fos immunoreactivity in the dorsal motor nucleus of the vagus (DMV) and nucleus tractus solitarius (NTS) (104). Furthermore, EA at PC6, primarily by inhibiting the transmission of gamma-aminobutyric acid (GABA) to the DMV, can alleviate the inhibition of efferent vagal motor fibers and thus promote efferent VN activity and gastric motility (105). The DMV and NTS exhibit commonality in their involvement with ST36 (106). EA at ST36 has been shown to increase c-Fos expression in DMV neurons and promote gastric myoelectric activity, which is regulated by VN (107). ST36′s effects are contingent on the VN, and EA at ST36 has been shown to enhance gastric myoelectric activity in rats (108). EA at ST36 can activate different adrenergic receptors through the spinal afferent-brainstem-vagus efferent-neuropeptide Y (NPY) + adrenal chromaffin cell-norepinephrine (NE) pathway to inhibit inflammation (109). The studies reported in the literature we cited above corroborate that the international academic community in this area has recognized the efficacy of acupuncture in NVP/HG treatment, which is supported by strong evidence-based medical data. Taken together, these studies suggest that the vagus nerve is significantly involved in EA at PC6 and ST36, and that its regulation may explain the mechanism by which acupuncture treats NVP or HG.

## 4 Vagus nerve stimulation

The VNS has garnered increased attention from researchers and practitioners in the medical community as a potential therapeutic intervention for various diseases. The early VNS device required surgical implantation of an electrode around the left vagus nerve, making it an invasive procedure (110). In recent years, non-invasive vagus nerve stimulation devices (nVNS), such as GammaCore and NEMOS, have emerged as significant areas of interest and are increasingly used for treating patients because of their relative safety and tolerance (111). GammaCore is a handheld and independent nVNS device authorized by the FDA. By directly touching the cervical skin surfaces, it delivers electrical signals to the vagus (112). NEMOS is an external device that provides transcutaneous VNS (ta-VNS) using a dedicated intra-auricular electrode (like an earphone) that stimulates the auricular branch of the VN (113). The pathogenesis of HG involves multiple systemic interactions, with the vagus nerve serving as the core pathway connecting the central nervous system, gastrointestinal tract, and placenta. In addition, vagus nerve stimulation mediates GDF15 signaling, gastrointestinal motility disorders, and anti-inflammatory and psychological stress responses in NVP/HG treatment.

### 4.1 Vagus nerve regulation of gastrointestinal motility in NVP/HG

The VN can mediate information transmission between the central nervous system (CNS) and the stomach. Tong et al. (114) found that the VN was most densely distributed in the stomach. After receiving VN signals, the NTS serves as the primary lower center for visceral primary sensory processing, except for the pelvic organs. Sensations such as the gastric stretch reaction and fullness can be transmitted to the NTS through the VN (115). Additionally, the dominant gastric vagal efferent fibers, which carry parasympathetic motor signals to the gastrointestinal (GI) tract, originate in the dorsal motor nucleus of the vagus (DMV), reinforcing VN's critical role in GI regulation (116). DMV and NTS together constitute the dorsal vagal complex (DVC), integrating visceral sensory afferent signals and gut parasympathetic preganglionic efferent signals (117). Taken together, these factors play a pivotal role in the regulation of gastrointestinal functional activities. The dense innervation of the VN in the gastrointestinal system is widely acknowledged for its regulatory role in gastric emptying, peristalsis, and gastric acid secretion, as well as in the excitation and inhibition of intestinal function (118, 119). A preliminary rodent study confirmed that 25 Hz VNS using optimal parameters was effective in improving gastric dyskinesia and promoting gastric emptying via the vagal-cholinergic pathway, suggesting that VNS may have therapeutic potential for functional gastrointestinal disorders (119). High-intensity EA in the deep tissue of ST36 in mice significantly promotes gastric motility, and the effect is entirely dependent on the vagal pathway (120). In depressive-like mice, electroconvulsive therapy enhances distal colonic motility via the subdiaphragmatic vagus nerve, which is abolished by vagotomy (121). Additionally, motilin-stimulated feeding is linked to gastric motility through the vagus nerve and increased c-Fos expression in tyrosine hydroxylase (TH) neurons in the AP and NST of the brain stem, as well as in activated neuropeptide Y and TH neurons in the arcuate nucleus of the hypothalamus (122). Electrical cervical vagus nerve stimulation (cVNS) affects gastric motility via slow gastric waves (123). EA provides a novel molecular mechanism for improving gastrointestinal motility in diabetic gastroparesis (DGP) via peripheral stimulation (ST36), spinal afferent (L4–L6), brainstem integration (NTS), and vagal efferent (gastric) circuits (124). Collectively, these findings demonstrated that VNS alleviates HG/NVP by normalizing gastrointestinal motility.

### 4.2 Vagus nerve alleviates NVP/HG by regulating the markers (GDF15) of genes and placenta

Evidence suggesting that GDF15 is a primary cause of hyperemesis gravidarum indicates that therapies targeting this pathway could be effective in treating this condition. Area postrema (AP) is anatomically linked to and interacts with the NTS (125). There has been an increasing amount of evidence suggesting that AP is a target site for the signaling marker GDF15 (126–129). The DMV, which, in turn, cooperates with the AP and NTS, forms the DVC (130, 131). According to Tsai et al. (13), the effect of GDF15 depends on the action of AP/NTS, and the absence of AP/NTS reduces the effectiveness of GDF15 treatment. GDF15 is also a nonspecific blood biomarker of HG, which crosses the blood-brain barrier to bind to the glial cell-derived neurotrophic factor family receptor α-like (GFRAL) receptor in the area postrema (AP) and nucleus of the solitary tract (NTS) of the brainstem to influence food intake, nausea, body weight, and insulin sensitivity (13, 14). Critically, activated AP/NTS neurons project to the hypothalamic arcuate nucleus (ARC), where vagal efferent signaling reduces gastric motility (17). TaVNS promotes the release of acetylcholine (ACh) to improve placental function (132). Maternal VNS treatment is safe during pregnancy and ameliorates L-NAME-induced preeclampsia-like symptoms in rats through inhibition of the inflammatory response (133, 134). The above literature suggests that the vagus nerve targets the AP/NTS of the DVC to modulate the GDF15 signaling, which may alleviate NVP/HG.

### 4.3 Vagus nerve regulation of *H. pylori*-related inflammation in NVP/HG

Treating and eradicating *H. pylori* can alleviate nausea and vomiting during pregnancy (135). *H. pylori* infection may contribute to an imbalance in the human gastrointestinal flora. “The gut-brain axis” is composed of multiple components, including the VN, immune system, and bacterial metabolites and products (136). Substantial evidence has demonstrated that VNS can ameliorate pro-inflammatory effects induced by gut dysbiosis and modulate immune functions (137). VN inhibits the production of pro-inflammatory cytokines that constitute the cholinergic anti-inflammatory pathway (138). A systematic review and meta-analysis of various VNS methods, including transcutaneous auricular VNS (taVNS), transcutaneous cervical VNS (tcVNS), invasive cervical VNS (iVNS), and electroacupuncture VNS (eaVNS), indicated the ability of VNS to modulate inflammatory markers such as C-reactive protein (CRP), interleukin (IL)-10, and interferon (IFN)-γ (139). The VN, therefore, exerts potent anti-inflammatory effects (118). Inhibition of enhanced efferent vagus nerve activity can counteract oxidative stress/inflammation/apoptosis/autophagy signaling involving *H. pylori* (140). In fact, vagus nerve stimulation has been shown to alleviate symptoms of inflammatory bowel disease (IBD) via the inflammatory reflex to reduce cytokines and colonic inflammation (141). Additionally, vagus nerve stimulation (VNS) has been demonstrated to reduce the inflammation induced by endotoxemia and decrease gut permeability (142, 143). Beyond local gut functions, microbiota influence stress responses and neurological health via inflammasome-derived cytokines, linking gut-derived signals to systemic diseases through the vagus nerve and the HPA axis (144). We, therefore, propose that the vagus nerve may represent a potential therapeutic agent for HG/NVP by regulating the intestinal flora and immune function, thereby exerting anti-inflammatory effects against *H. pylori*.

### 4.4 Vagus nerve regulation of psychosocial factors in NVP/HG

Studies on both rodents and humans have demonstrated that VNS can mitigate mental disorders such as anxiety and depression (145–149). Electrical stimulation of the cervical VN is an FDA-approved therapy for depression (150). Recent studies have confirmed that symptoms of depression can increase the risk of dysfunction of the hypothalamus-pituitary-adrenal (HPA) axis (151). Notably, VNS can attenuate dysfunction in the HPA axis (152, 153). Subdiaphragmatic vagotomy reversed behavioral changes suggestive of depression and anxiety in rats with functional dyspepsia (FD) (154). VN signaling can modulate depressive-like behaviors by communicating both the pro- and antidepressant effects of gut molecules to the brain, while loss of gut-originating vagal signaling also has anxiolytic effects (155). Clinical trials conducted in individuals suffering from major depressive disorder (MDD) have revealed that transcutaneous VNS (tVNS) is effective in reducing depressive symptoms (156, 157). The tVNS reduces reactivity to emotionally charged stimuli in MDD (158). The gain of vagal function with stimulation of the left vagus nerve at the cervical level in male rats reduces anxiety-like behaviors in the exploration of open areas in the elevated plus maze (EPM) (148). Given the contraindications for psychotropic drugs during pregnancy, we propose VNS as a safe neuromodulatory intervention for HG/NVP that normalizes stress responses.

## 5 VNS-targeted electroacupure at auricular/cervical points: a novel therapeutic model for NVP/HG

In fact, both acupuncture and VNS are auxiliary alternative physical therapies that act on the human body, with mechanisms that can serve as references for treatment approaches. EA, which combines acupuncture with electrophysiological techniques, is commonly used in clinical practice and in basic research. It is not only widely used in TCM to treat diseases but has also been recommended by the World Health Organization (WHO) and the National Institutes of Health (NIH) as surface stimulation therapy (159, 160). The biological mechanism often involves regulation of the autonomic nervous system (ANS). ANS can interconnect external somatosensory inputs with internal organ responses via the central nervous system (CNS) (161). According to the principle of anatomical acupoint selection, the VN in the auricular and cervical regions is also the preferred site for VNS. Whether stimulating the VN or inhibiting the sympathetic nerve, EA at acupuncture points in the auricular and cervical regions can regulate the VN more notably than traditional acupuncture (162, 163), especially when the latter stimulates PC6 in the upper limb and ST36 in the lower limb. Despite this mechanistic synergy, few studies have addressed vagus nerve-targeted acupoint selection in NVP/HG. This lack of literature underscores the gap and pioneering nature of employing the vagus nerve as a preferred acupuncture point for addressing these conditions. For pregnant patients, therapeutic safety is paramount. Our clinical experience demonstrates that electroacupuncture at cervical/auricular acupoints safely and effectively treats sudden sensorineural hearing loss in the first trimester of pregnancy (164). VNS serves as a viable therapeutic alternative or adjunct for managing gestational headaches (165). In emergency department management of supraventricular tachycardia (SVT) with stable hemodynamics during pregnancy, VNS should be considered (166). VNS is a promising, feasible, and effective intervention for antenatal depression (167). Although the vagus nerve interfaces with reproductive pathways, current evidence suggests that VNS remains relatively safe and effective for both the mother and fetus during pregnancy (168). To conclude, we propose that EA at auricular/cervical acupoints may effectively regulate vagal signaling, thus providing enhanced therapeutic efficacy against NVP/HG.

## 6 Conclusion and future directions

**Figure 1:**
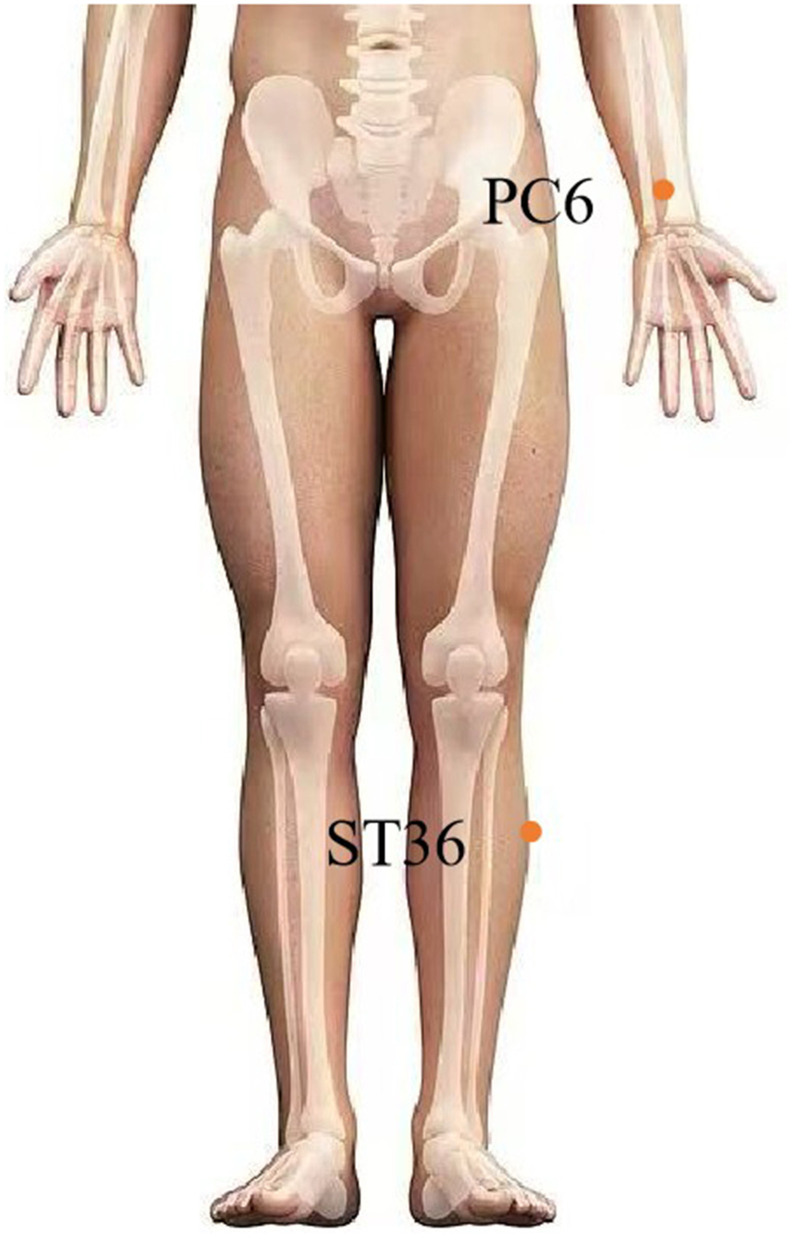
EA at classic acupoints for NVP/HG management.

**Figure 2:**
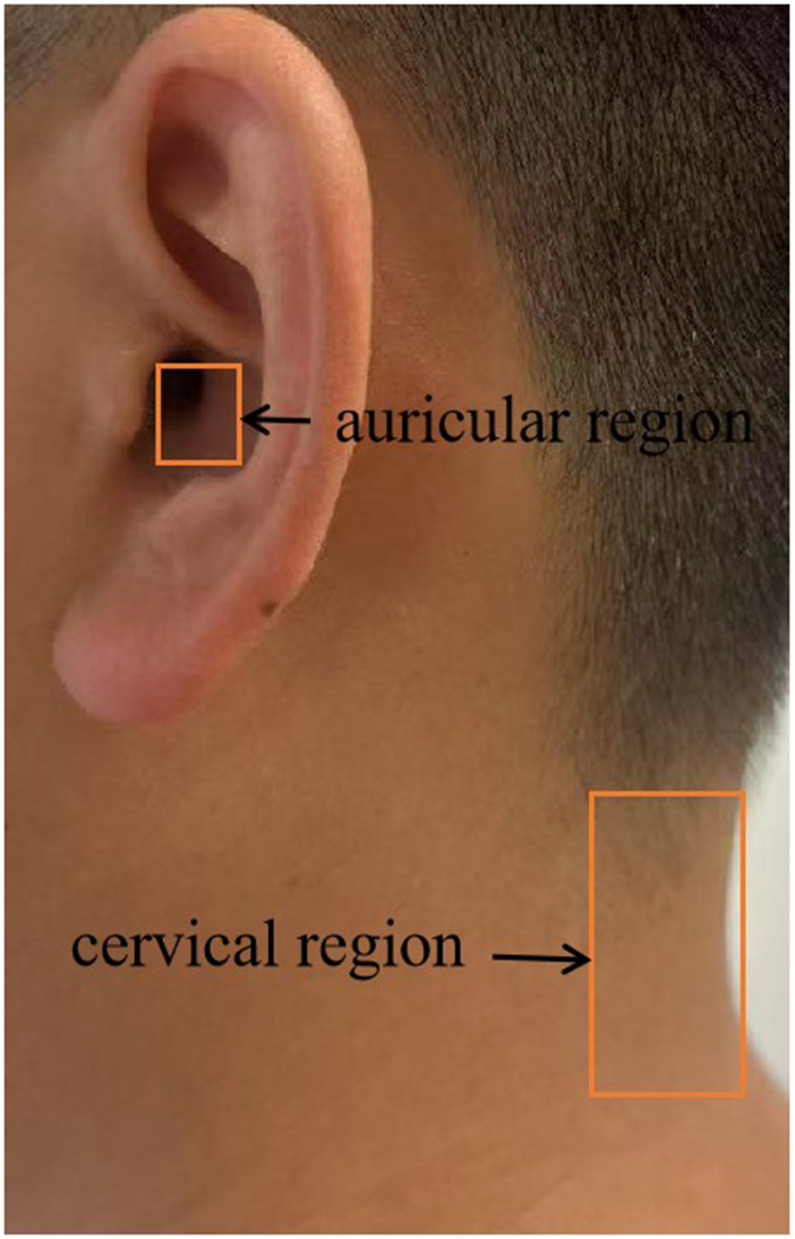
VNS/EA at novel anatomical sites for NVP/HG management.

HG represents a severe pathological form of NVP for which no definitive or permanent cure currently exists. Bottom of FormGiven this, physicians focus on providing treatments that alleviate symptoms and offer supportive care. As the disease burden of severe NVP and HG is largely beyond the estimation, developing novel therapeutics for gravidas with NVP or HG is of vital importance. The current review provides the strongest evidence-based data and literature that confirms the effectiveness of EA in treating NVP/HG by selecting PC6 and ST36 as core acupuncture points that are closely linked to the vagus nerve. Neurophysiologically, this review, based on an extensive literature search and review of current studies, proposes that acupuncture can significantly alleviate NVP/HG through modulation of the vagus nerve by stimulating local acupuncture points. Despite extensive research on the VNS, few investigations have been conducted on its use for the treatment of NVP/HG. Studies have confirmed that the VNS can regulate the pathogenesis of NVP/HG, as revealed in our review. Therefore, we propose a VNS-based model, which acts on multiple possible pathways for alleviating NVP/HG by (1) attenuating disruptions in gastrointestinal tract movement and function, (2) managing markers (e.g., GDF15/MIC-1) of genes and placental function, (3) regulating *Helicobacter pylori*, and (4) managing psychosocial factors such as anxiety and depression. However, there exists scant literature that considers the location of the vagus nerve as a site for acupuncture points for treating the NVP/HG. Compared to PC6 and ST36, anatomically speaking, since the VNS regulates primarily the vagus nerve in the cervical and/or auricular regions, we propose that electroacupuncture in the cervical and auricular regions can more effectively regulate the vagus nerve and alleviate the NVP/HG (Figures 1, 2). Therefore, electroacupuncture at the cervical and auricular points to regulate the vagus nerve may be a novel therapeutic approach with great potential for treating NVP/HG.
